# Ectopic Colonization and Immune Landscapes of Periodontitis Microbiota in Germ-Free Mice With Streptozotocin-Induced Type 1 Diabetes Mellitus

**DOI:** 10.3389/fmicb.2022.889415

**Published:** 2022-06-10

**Authors:** Xin Shen, Hong Wei, Jian Li, Wei Wei, Bo Zhang, Changqing Lu, Caixia Yan, Shuzhen Li, Lirong Bao, Jinmei Zhang, Cheng Zhang, Yan Li

**Affiliations:** ^1^State Key Laboratory of Oral Diseases, National Clinical Research Center for Oral Diseases, West China Hospital of Stomatology, Sichuan University, Chengdu, China; ^2^Central Laboratory, Clinical Medicine Scientific and Technical Innovation Park, Shanghai Tenth People’s Hospital, Tongji University, Shanghai, China; ^3^Institute of Immunology, PLA, Army Medical University, Chongqing, China; ^4^Department of Stomatology, Minda Hospital of Hubei Minzu University, Enshi, China; ^5^Department of Anatomy, West China School of Basic Medical and Forensic Medicine, Sichuan University, Chengdu, China; ^6^CAS Key Laboratory of Environmental Biotechnology, Research Center for Eco-Environmental Sciences, Chinese Academy of Sciences, Beijing, China

**Keywords:** periodontitis microbiota, type 1 diabetes mellitus, ectopic colonization, immune regulation, germ-free mice

## Abstract

A two-way relationship between diabetes and periodontitis has been discussed recently. Periodontitis microbiota might affect the immune homeostasis of diabetes, but the molecular mechanism of their interactions is still not clear. The aims of this study were to clarify the possible immune regulatory effects of periodontitis microbiota on diabetes and the correlation between immunomodulation and ectopic colonization. A model of germ-free mice with streptozotocin-induced type 1 diabetes mellitus (T1D), which was orally inoculated with mixed saliva samples for 2 weeks, was used in this study. Those mice were randomly divided into two groups, namely, SP (where the T1D mice were orally inoculated with mixed saliva samples from periodontitis patients) and SH (where the T1D mice were orally inoculated with mixed saliva samples from healthy subjects). Ectopic colonization of saliva microbiota was assessed using culture-dependent method and Sanger sequencing, and the composition of gut microbiota was analyzed using 16S rRNA gene sequencing. Changes in 15 types of immune cells and six cytokines either from the small intestine or spleen were detected by multicolor flow cytometry. The correlation between gut microbiota and immune cells was evaluated by redundancy analysis. Although periodontitis microbiota minorly colonized the lungs, spleens, and blood system, they predominantly colonized the gut, which was mainly invaded by *Klebsiella*. SH and SP differed in beta diversity of the gut bacterial community. Compared to SH, microbial alteration in small intestine occurred with an increase of *Lacticaseibacillus*, *Bacillus*, *Agathobacter*, *Bacteroides*, and a decrease of *Raoultella* in SP. More types of immune cells were disordered in the spleen than in the small intestine by periodontitis microbiota, mainly with a dramatical increase in the proportion of macrophages, plasmacytoid dendritic cells (pDCs), monocytes, group 3 innate lymphoid cells, CD4-CD8- T cells and Th17 cells, as well as a decline of αβT cells in SP. Cytokines of IFNγ, IL17, and IL22 produced by CD4 + T cells as well as IL22 produced by ILCs of small intestine rose in numbers, and the intestinal and splenic pDCs were positively regulated by gut bacterial community in SP. In conclusion, periodontitis microbiota invasion leads to ectopic colonization of the extra-oral sites and immune cells infiltration, which might cause local or systemic inflammation. Those cells are considered to act as a “bridge” between T1D and periodontitis.

## Introduction

Diabetes is a metabolic disease of high prevalence, with 150 million people affected worldwide, which is divided into two main types, namely, type 1 diabetes mellitus (T1D) and type 2 diabetes mellitus (T2D). T1D is a T cell mediated autoimmune disease accounting for 10–15% of all diabetes cases, which is caused by destruction of pancreatic β cells and results in hyperglycemia (Katsarou et al., 2017). Periodontitis, initiated by microbial dysbiosis and manifested in periodontal tissue destruction and tooth loss, has been identified as one of the sixth most crucial complications in diabetes (Graves et al., 2019). Chronic diseases cause a significant burden on global public health services (Pontes Andersen et al., 2007). At present, although a two-way relationship between diabetes mellitus and periodontitis has been discussed (Babatzia et al., 2020), the exact molecular mechanism of how periodontitis microbiota took part in the progression of T1D remains unclear.

To recognize and defend against pathogens, the innate immune system is the first line, to react rapidly with multiple cells, including macrophages (MFs) and dendritic cells (DCs), each type of cells having the specific functions. As the second line of host defense, the adaptive immune response mainly consists of T and B cells, which bind to receptors of the antigen-presenting cells to induce inflammatory cytokines and antibodies. CD4 + T cells (Th1, Th17, Th22, and Tregs) and CD8 + T cells are the most important subsets of T cells. Both innate and adaptive immune cells are involved in the different stages of T1D pathogenesis, which are populated by CD8 + T cells, CD4 + T cells, Tregs, B cells, DCs, and MFs (Lehuen et al., 2010; Ferretti and La Cava, 2016). Researchers showed that both splenic CD4 + and CD8 + T cells are essential for the development of T1D mice (Phillips et al., 2009). In addition to the role in the pathogenesis of T1D, T cells may also help to prevent β-cell destruction. There is strong evidence that Tregs are important to prevent T1D in non-obese diabetic mice. T1D leads to a reduction in Tregs, in conjunction with an expansion of Th17 cells (Salomon et al., 2000; Shao et al., 2012). Moreover, as for the role in presenting antigens, B cells play a pathogenic effect on T1D onset (Mariño et al., 2011). When the adaptive immune responses become dysregulated, there are typically multiple abnormalities in the innate immune system that precede them. MFs and DCs might also play a pathogenic role in initiation and destruction phases of T1D by facilitating differentiation of CD8 + T cells (Lehuen et al., 2010).

Various genetic and environmental factors can lead to T1D by compromising the immune system. However, 85% cases of T1D occur in patients without a family history of diabetes (Gianchecchi and Fierabracci, 2015). It is pointed out that the environmental factors become more important in the pathogenesis of T1D, especially in terms of microorganisms. Recently, the interactions of microbiota and immune system have been widely involved in T1D onset (Siljander et al., 2019; Dedrick et al., 2020). The local and systemic thresholds in activation of immune cells are controlled by the gut resident commensals. Microbes residing in the other barrier sites, such as oral cavity, may also contribute to regulate the local immune responses of tissues. Impaired immune responses and dysbiosis of gut microbiota induced by the periodontal pathogens might affect the insulin metabolism in T1D or T2D mice. For example, the possible effect of *P. gingivalis* on the gut immune system is that composition shifts in the gut microbiota trigger the pathogenic T cells responses (Blasco-Baque et al., 2017; Ohtsu et al., 2019). Nevertheless, there is still not enough evidence to clarify the overall immunoregulation effect of periodontitis microbiota on T1D development. Moreover, the bacteria that penetrate the mucosal barrier into circulatory system might be one mechanism of periodontal pathogens affecting diabetes (Kocher et al., 2018). The plasma glucose control is improved by periodontal treatment by removing the bacterial biofilm (Mauri-Obradors et al., 2018). However, available relationships between periodontal bacteria and diabetes were mostly reported in T2D. Studies of how the periodontal bacteria affect T1D are still limited. It is necessary to determine the exact factors in periodontitis microbiota that cause dysregulation of the immune response in T1D onset.

Therefore, in this study, the ectopic colonization ability in the extra-oral sites and the local and systemic immunoregulation of periodontitis microbiota in T1D onset are broadly studied. It is hypothesized that periodontitis microbiota may directly translocate to distant organs, which abnormally activates the immune responses and eventually leads to the accelerated development of T1D.

## Materials and Methods

### Collection of Saliva Samples

This study was authorized by the ethics committee of West China Hospital of Stomatology, Sichuan University (WCHSIRB-D-2017-035). Informed consent was provided by all donors. All saliva samples were collected at the Periodontology Department of the West China Hospital of Stomatology, according to the methods described earlier (Miller et al., 2021). The patients were diagnosed with periodontitis according to the clinical classification and definition of periodontitis (Tonetti et al., 2018). Donors with systemic diseases, including diabetes, a history of smoking, a recent history (3 months) of antibiotic use or the periodontitis treatment were excluded. Eight adult volunteers with periodontitis (four women, four men, age 44–72 years) were selected; and 12 healthy donors of matched gender and age with periodontitis patients were included as the control group. All saliva samples were mixed into samples of equal volume (5 ml) as the representatives of each group, verified by 16S rRNA gene sequencing.

### Animals and Treatments

This study was authorized by the ethics committee of West China Hospital of Stomatology, Sichuan University (WCHSIRB-D-2017-069). A total of 12 germ-free BALB/c mice (female mice, 5 weeks old) provided by the Department of Laboratory Animal Science, College of Basic Medical Sciences, Army Medical University (Chongqing, China) were used. Streptozotocin (STZ, Sigma-Aldrich United States) at a dose of 50 mg/kg was injected intraperitoneally into germ-free mice (*n* = 8) for the five consecutive days as previously described (Chen et al., 2015). One week after the first injection, the fast plasma glucose level was detected to determine the success of model of germ-free mice with STZ-induced T1D before colonization of saliva samples. To ensure the sterile conditions without contamination, the feces of all mice were collected and cultured in the BHI media per week. After germ-free mice with STZ-induced T1D models were successfully established, all the mice were randomly divided into two groups, including the mice inoculated with mixed saliva samples from periodontitis patients (SP, *n* = 4), of which, one mouse died was excluded, and the mice inoculated with mixed saliva samples from healthy subjects (SH, *n* = 4). They were inoculated with a 200 μl mixture of fresh saliva by swabs without anesthesia, and the teeth of mice were swabbed for 1 min per mouse (Li et al., 2019). All operations were performed under the sterile conditions throughout the experiment. Untreated germ-free mice (GF, *n* = 4) were considered as the blank control. Each group of mice was housed in separate gnotobiotic isolators for 2 weeks, before being euthanized.

### Bacterial Culture, Isolation, and Identification

All suspensions of systemic organs, intestinal contents, and blood of equal quality or volume were collected and cultured on the plates of Brain Heart Infusion medium supplemented with hemin and vitamin K, under strictly anaerobic conditions (80% N_2_, 10% H_2_, 10% CO_2_) at 37°C for 3–7 days. Colony-forming units (CFUs) were counted for each plate. Bacterial DNAs were extracted from the representative colonies (the numbers of CFUs were above 10) according to the colony morphology and Gram staining, or all the colonies (the numbers of CFUs were below 10). Universal primers 27F and 1492R were used for PCR amplification and sent to Biochemical Bioengineering (Shanghai, China) for Sanger sequencing. Sequences were identified by a blast alignment in the NCBI database > 99.5% similarity (Sayers et al., 2010).

### Illumina Sequencing and Bioinformatics Analysis of 16S rRNA Gene Sequencing

Bacterial DNAs of the small intestinal contents were extracted using the QIAamp Fast DNA Stool Mini Kit (Cat No. 51604, TIANGEN Biotech, China) and the TIANamp Swab DNA Kit (Cat No. DP322, TIANGEN Biotech, China) was applied for saliva. F338 and R806 primers were used to amplify the bacterial 16S rRNA genes, which focused on regions of V3-V4 followed by Illumina HiSeq technology sequencing. All the pre-processing of sequences was conducted by an Galaxy-based pipeline in Denglab^1^ (Feng et al., 2017). The raw sequencing was quality-filtered by Trimmomatic (V0.33) and merged using FLASH (V1.2.11). Operational taxonomic units (OTUs) table was generated by UPARSE with a clustering threshold of 0.97. In total, 692,105 high-quality sequences were obtained. Silva database was used to annotate the taxonomic information (Quast et al., 2013). Rarefaction curves were analyzed by calculating the species richness of bacterial OTUs. The rarefied OTU table was applied for the most downstream analyses based on the sample with the least sequences (77,627 reads/per sample) and conducted on the website (see text footnote 1). Alpha-diversity indices (Simpson, Observed_richness, and Pielou_evenness) were estimated. Principal component analysis (PCA) was applied to determine the dissimilarity between bacterial communities of the two groups. Three different non-parametric analyses, namely, the multi-response permutation procedure (MRPP), permutational multivariate analysis of variance (PERMANOVA), and analysis of similarities (ANOSIM) were performed. The relative abundances of bacterial taxa at phylum, class, and genus levels were calculated.

### Lymphocytes Preparation and Multi-Color Flow Cytometry

The proportions of the following 15 immune cells were analyzed as follows: monocytes (Monos), MFs, mononuclear phagocytes (MNPs), CD11B + dendritic cells (CD11B + DCs), CD11B- dendritic cells (CD11B-DCs), pDCs, group 3 innate lymphoid cells (ILC3s), B cells, γδT cells (gdT), αβT cells (abT), CD4-CD8- T cells (DN), CD8 + T cells, CD4 + T cells, Th17, and Tregs. And the proportions of the following six cytokines were detected: IL17, IFNγ, IL22, and IL10 produced by CD4 + T cells, as well as IL17 and IL22 produced by ILCs. Single-cell suspensions of the small intestine and spleen were prepared as previously described (Benakis et al., 2016). Anti-mouse CD16/32 antibody was used for blocking the Fc domain before staining the surface or intracellular markers. All antibodies were divided into three groups and listed in Supplementary Table 1. To detect the innate immune cells, the first group included antibodies against CD45, CD19, Ly6c, PDCA-1, CD11c, CD11b, F4/80, and CD103. To detect the adaptive immune cells, the second group included antibodies against CD45, CD19, TCRß, TCRgd, CD4, CD8, Foxp3, and Rorγ. To detect the cytokines, the third group included antibodies against CD45, CD4, TCRß, TCRgd, IL17a, IFNγ, IL22, and IL10. Procedures of cell markers staining were following the instructions. BD Celesta was used to detect changes in cells. To ensure equal gating criteria and scoring, the raw data were independently analyzed by two individuals using the Kaluza software (version 2.1.1, Beckman Coulter, United States). Heatmap was drawn through the following steps: First, the fold change cell values in comparison of group GF in each cell type were log2 transformed; second, the values of each row were normalized to [−1, 1]; and finally, a package in R was used to draw the heatmap.

### Redundancy Analysis

Variance inflation factor (VIF) was applied to determine which cells were highly autocorrelated and cells with the VIF value above 10 were excluded to further analysis, and redundancy analysis (RDA) was adopted to analyze the correlation between geographical distribution of gut bacterial communities and immune cells in small intestine and spleen, using a package “vegan” in R version 3.6.2 (Sadyś et al., 2015). Envfit analysis in package “vegan” was applied to identify the significant immune cells affected by the microbial composition distribution.

### Statistical Analyses

Data were calculated as means ± SEM. All statistical analyses were performed using GraphPad Prism 6 software (GraphPad Prism6 Software, Inc., United States) or SPSS (version 24.0, IBM Corporation, United States). Wilcoxon rank-sum test was applied in taxonomy analysis at phylum, class, and genus levels. Two-tailed unpaired Student’s *t*-test was applied in difference alpha-diversity indices and the immune cells. *P* < 0.05 was considered significant.

## Results

### Ectopic Colonization Ability of Periodontitis Microbiota in the Extra-Oral Sites

**FIGURE 1:**
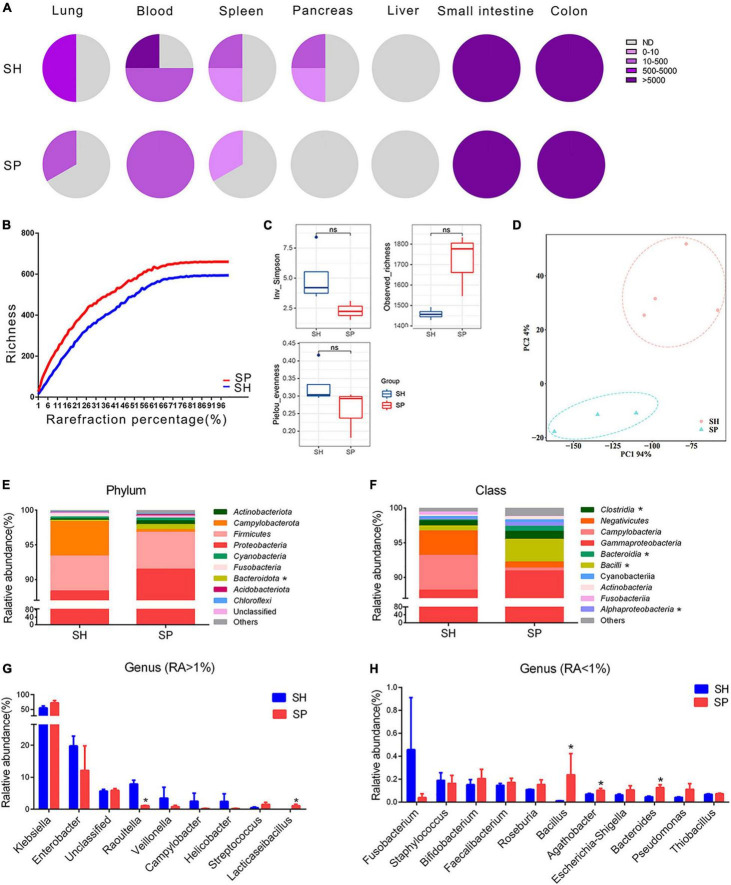
Ectopic colonization ability of periodontitis microbiota in the extra-oral sites. **(A)** Fan charts of CFU numbers cultured form the extra-oral sites. **(B)** Rarefaction curves based on the Chao1 richness. **(C,D)** Alpha and beta diversity analyses in the gut microbiota. **(E–H)** Relative abundances of phylum (top 10), class (top 10), and genus (top 20) level in the gut microbiota. Data were calculated as means ± SEM. Unpaired Student’s *t*-tests were performed for panel **(C)**. MRPP, PERMANOVA, and ANOSIM were performed for panel **(D)**. The Wilcoxon rank-sum test was performed for panels **(E–H)**. Significant results when: *p < 0.05. SH, germ-free mice with STZ-induced T1D that orally inoculated with mixed saliva samples from the healthy (*n* = 4), SP, germ-free mice with STZ-induced T1D that orally inoculated with mixed saliva samples from the periodontitis (*n* = 3).

Periodontitis microbiota primarily colonized the small intestines and colons, followed by the lung, blood, and spleen of germ-free mice with STZ-induced T1D. Neither pancreas nor liver could be colonized by periodontitis microbiota (Figure 1A and Supplementary Figure 1H). Sanger sequences were blasted in NCBI website to identify the closely related species, and those with a more than 99.5% similarity are summarized in Table 1. Compared to SH, the diversity of the species was decreased in SP group, and the main bacteria identified were *Klebsiella pneumoniae* in the extra-oral sites.

**TABLE 1:** Identification of bacteria translocating into the extra-oral sites between SH and SP.

Organs	Closest species	*CFUs Positive	Closest species	*CFUs Positive
	SH		SP	
Lung^Δ^	*Klebsiella pneumoniae*	2/7	*Klebsiella pneumoniae*	1/3
	*Staphylococcus aureus*	1/7	*Kelbsiella aerogenes*	1/3
	*Kelbsiella aerogenes*	3/7	*Bacterium RB5-FF-23*	1/3
	*Raoultella ornithinolytica*	1/7		
Blood^Δ^	*Klebsiella pneumoniae*	1/10	*Klebsiella pneumoniae*	7/10
	*Staphylococcus aureus*	3/10	*Enterobacter hormaechei*	2/10
	*Kelbsiella aerogenes*	5/10	*Aerococcus viridans*	1/10
	*Raoultella ornithinolytica*	1/10		
Pancreas	*Staphylococcus aureus*	5/8	/	0/0
	*Staphylococcus epidermidis*	1/8		
	*Kelbsiella aerogenes*	2/8		
Spleen	*Staphylococcus aureus*	1/3	*Klebsiella pneumoniae*	1/1
	*Kelbsiella aerogenes*	1/3		
	*Staphylococcus saccharolyticus*	1/3		

*CFUs positive/total CFUs. ^Δ^Owing to large numbers of CFUs existed in SH, the representative CFUs were selected for Sanger sequencing, according to the colony morphology and results of Gram staining.

Illumina HiSeq technology was applied to determine the bacterial community from seven samples of the small intestinal contents. In total, 692,105 high-quality sequences were obtained with an average length of 470 bp and an average of 98,872 sequences were generated per sample. The Silva database was used to categorize these sequences into 38 classified phyla, 438 genera, and 2,342 OTUs. The rarefaction curves tended to be flat in two groups (Figure 1B). The dissimilarity test showed significant differences by three parameters (MRPP = 0.65, *p* = 0.03; PERMANOVA = 1.11, *p* = 0.05; and ANOSIM = 0.87, *p* = 0.03) between SP and SH. However, the data in alpha diversity indices of the Inv_Simpson (*p* > 0.05), Pielou_evenness (*p* > 0.05) and Observed_richness (*p* > 0.05) indicated a similar bacterial community between two groups (Figure 1C). PCA was conducted to visualize the different diversities of microbiota in two groups (Figure 1D). *Proteobacteria* (90%) were the dominant phylum in the small intestine of two groups. Compared to SH, the relative abundance of *Bacteroidota* was increased in the top 10 of phylum level (*p* < 0.05), and the ratio of *Firmicutes* to *Bacteroidetes* was nearly five times lower in SP (Figure 1E). At the relative abundance of top 10 class level, four types were significantly different between SH and SP, including *Clostridia*, *Bacteroidia*, *Bacilli*, and *Alphaproteobacteria* (*p* < 0.05) (Figure 1F). *Klebsiella* were the dominant genera in SH and SP, accounting for nearly 55% and 72%, respectively. Among genera of the relative abundance above 1%, *Raoultella* (1.06%, *p* < 0.05) were decreased for seven folds and *Lacticaseibacillus* (1.10%, *p* < 0.05) were increased in SP (Figure 1G). As the minor components, several genera with relative abundances less than 1% were significantly different in the two groups, including *Bacillus*, *Agathobacter*, and *Bacteroides* in terms of the relative abundance of top 20 genus level (Figure 1H).

The success of animal models was verified in this study. The fast plasma glucose concentrations of the STZ-treated germ-free mice were 10.55 ± 2.92 mM, while the control group remained normoglycemic, at 6.85 ± 0.42 mM with a significant difference (Supplementary Figure 1A). Namely, the model of germ-free with STZ-induced T1D was constructed successfully. Before bacterial colonization, the diversity of microbial community of the mixed saliva samples was evaluated. 16S rRNA gene sequencing was conducted for the mixed saliva samples from eight periodontitis patients and 12 healthy volunteers (Supplementary Figure 1B). The bacterial distribution was dramatically different in the genus level between periodontitis patients and healthy volunteers. Specifically, the former group was dominated by *Porphyromonas, Fusobacterium*, and *Treponema*, which were the red or yellow complex in the periodontitis. At the end point of experiment, the ceca in SH and SP were much smaller than those in group GF (Supplementary Figure 1C), indicating that the oral bacteria successfully translocated into the gut. The pancreatic tissues were collected and then stained with hematoxylin-eosin staining. Compared to the GF group, the pancreatic islets were emptied with vacuolar degeneration, necrosis, and disappearance of pancreatic β cells (indicated by a black arrow in Supplementary Figure 1D). Vascular dilatation and congestion of the intra and para-pancreatic islets were observed (indicated by a blue arrow in Supplementary Figure 1D). The effects of periodontitis microbiota on the fast plasma glucose and fast plasma insulin were also investigated (Supplementary Figures 1E,F). Compared to SH, there were no significant changes in either of the two parameters in SP. Moreover, alveolar bone loss was not induced by transferring periodontitis bacteria to germ-free mice with STZ-induced T1D (Supplementary Figure 1G).

### Immunological Changes of the Small Intestine in Response to Colonization of Periodontitis Microbiota

**FIGURE 2:**
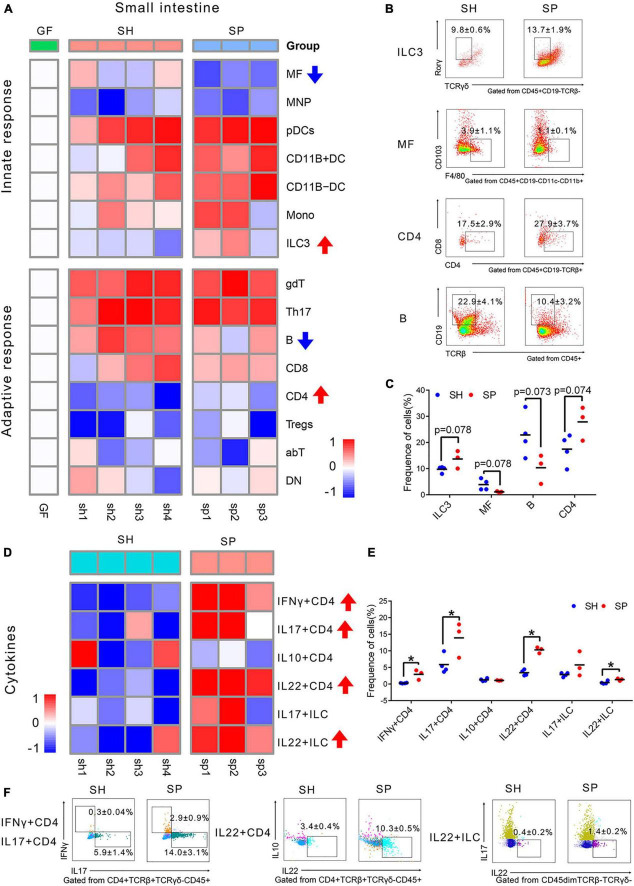
Immune cells changes in the small intestine induced of periodontitis microbiota colonization. **(A)** Heatmap of 15 immune cells in the small intestine. Compared to SH, the frequency of immune cells was rising, as labeled by red arrows, while those of immune cells was decreased, as labeled by blue arrow in SP. **(B)** Representative flow cytometry dot plots of ILC3s, MFs, B cells, and CD4 T cells. **(C)** Histogram of ILC3s, MFs, B cells, and CD4 + T cells. **(D)** Heatmap of six cytokines in the small intestine. **(F)** Histogram of cytokines. **(E)** Representative flow cytometry dot plots of IFNγ^+^ CD4, IL17^+^ CD4, IL22^+^ CD4 T cells, and IL22^+^ ILCs. Data were calculated as means ± SEM. Unpaired Student’s *t*-tests were performed on all the data. The mean values of the four mice in group GF represented by the one square, while the individual value of each mouse in SH and SP represented by the one square. GF, germ-free mice (*n* = 4). Significant results when: *p < 0.05.

A total of 15 types of immune cells were chosen, including seven innate and eight adaptive immune cells, to investigate how periodontitis microbiota affected local immunity in the small intestine (Supplementary Table 2 and Supplementary Figure 2A). The gating strategy for the two staining panels, namely, innate immune cells (Supplementary Figure 2B) and adaptive immune cells (Supplementary Figure 2C), was demonstrated by representative flow cytometry plots. A total of 195 independent immunophenotypes triggered by oral saliva microbiota in the small intestine were generated. Normalized fold changes relative to the group GF were described in a heatmap (Figure 2A).

As shown in the heatmap, SH and SP shared a similar immune landscape in the small intestine. Compared to SH, there were fewer responsive immune cells of the small intestine in SP, with only four types of cells being different. The proportions of ILC3s (*p* = 0.078) and CD4^+^ T cells (*p* = 0.074) were rising in SP, concomitant with a decline of MFs (*p* = 0.078) and B cells (*p* = 0.073). The most notable changes were considered as MFs (reported as% of CD45^+^CD19^–^ cells) and B cells (reported as% of CD45^+^ cells), which were strongly affected by periodontitis microbiota. The frequency of MFs was decreased from 3.9 ± 1.1% to 1.1 ± 0.1%, with nearly three-fold changes. A more than two-fold decrease was shown in the proportion of B cells in SP, from 22.9 ± 4.1% to 10.4 ± 3.2% (Figures 2B,C). However, most of the remaining immune cells did not show significant changes (Supplementary Figures 3A,B).

Cytokines are an important factor for the development and activation of immune cells. Six cytokines were detected to uncover how the function of immune cells changed in the small intestine. CD4 + T cells producing IFNγ, IL17, IL10, and IL22 as well as ILCs producing IL17 and IL22 were included. The gating strategy for cytokines is shown in Supplementary Figure 2B and Supplementary Table 2. Compared to SH, CD4 + T cells were significantly activated by periodontitis microbiota, with the accumulation of IFNγ^+^ CD4 (*p* < 0.05), IL17^+^ CD4 (*p* < 0.05), and IL22^+^ CD4 (*p* < 0.05) T cells in the small intestine (Figures 2D–F). Moreover, the frequency of IL22 (*p* < 0.05) produced by ILCs was rising in SP. But, the frequencies of IL10^+^ CD4 (*p* > 0.05) and IL17^+^ ILC (*p* > 0.05) were not changed significantly in SP (Supplementary Figure 3C).

### Immunological Changes of the Spleen in Response to Colonization of Periodontitis Microbiota

**FIGURE 3:**
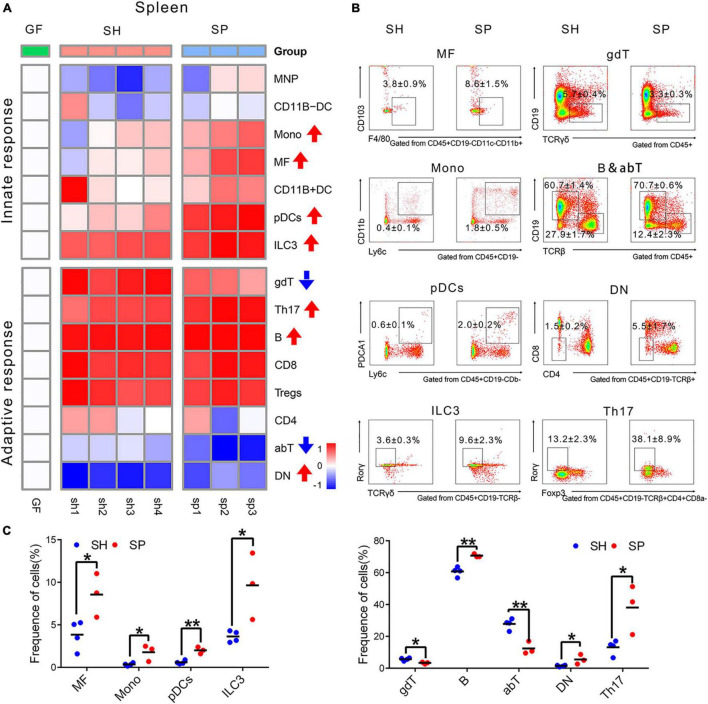
Immune cells changes in the spleen induced of periodontitis microbiota colonization. **(A)** Heatmap of 15 immune cells in the spleen. **(B)** Representative flow cytometry dot plots of immune cells with a significant difference. **(C)** Histogram of immune cells with a significant difference. Data were calculated as means ± SEM. Unpaired Student’s *t*-test were performed on all the data. Significant results when: *p < 0.05; **p < 0.01.

The same types of immune cells were detected to investigate how periodontitis microbiota affected the systemic immune response in the spleen (Figure 3A). Compared to SH, a total of four innate immune cells and five adaptive ones were significantly altered in SP. In terms of the innate immune response in the spleen, most of the cells appeared to be more responsive than those in the small intestine, demonstrated by modest changes in the proportion of immune cells. The proportions of MFs (*p* < 0.05), ILC3s (*p* < 0.05), Monos (*p* < 0.05), and pDCs (*p* < 0.05) were increased in SP, of which proportions of MFs and pDCs were strongly changed by more than threefold. The frequency of Monos increased from 0.4 ± 0.1% to 1.8 ± 0.5% combined with a rising proportion of pDCs from 0.6 ± 0.1% to 2.0 ± 0.2% (Figures 3B,C). Regarding adaptive immune response, a marked increase in the frequencies of B (*p* < 0.05), Th17 (*p* < 0.05), and DN cells (*p* < 0.05) was induced by periodontitis microbiota, as well as a decrease in those of αβT (*p* < 0.05) and γδT (*p* < 0.05). It occurred dramatically in the proportion of Th17 (reported as% CD4^+^CD8a^–^TCRb^+^CD19^–^CD45^+^ T cells) and DN cells (reported as% of TCRb^+^CD45^+^CD19^–^ cells), with a nearly three-fold increase, ranging from 13.2 ± 2.3% to 38.1 ± 8.9% and 1.5 ± 0.2% to 5.5 ± 1.7%, respectively (Figures 3B,C). However, the other cells were not significantly responsive to colonization of periodontitis microbiota (*p* > 0.05) (Supplementary Figures 4A,B).

### The Correlation Between the Local and Systemic Immune Cells and Gut Bacterial Community

**FIGURE 4:**
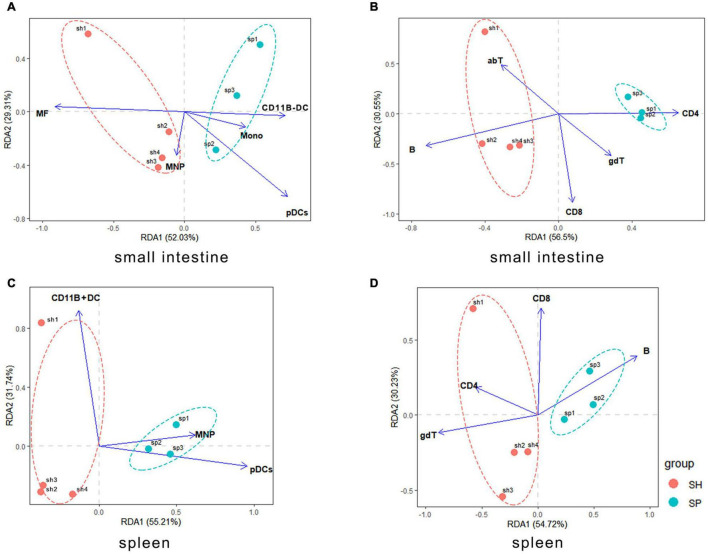
Correlations between the local and systemic immune cells and gut bacterial community. **(A,B)** Gut microbiota associated with the innate and adaptive immune cells in the murine small intestine. **(C,D)** Gut microbiota associated with the innate and adaptive immune cells in the murine spleen. The individual mouse gut microbial community was represented by one dot.

To determine whether the immunological changes in local and systemic organs were affected by the gut microbiota community that were colonized from periodontitis microbiota, RDA was performed. A total of 10 variables of the small intestine and seven cell types of the spleen were selected for further analysis in the RDA, of which the values of VIF were below 10 (Tables 2A,B). In both analyses of the small intestine and the spleen, nearly 55% and 30% of the variance at the first and second RDA axes were explained, respectively (Figure 4).

**TABLE 2A:** Results of VIF analysis in gut.

Gut	Mono	MF	MNP	CD11B-DC	pDCs	gdT	B	abT	CD4	CD8
VIF	2.5	4.9	3.9	3.1	5.7	4.3	2.2	2.8	3.1	3.2

**TABLE 2B:** Results of VIF analysis in spleen.

Spleen	CD11B + DC	MNP	pDCs	gdT	B	CD4	CD8
VIF	1.8	4.6	4.4	3.0	3.0	1.4	1.3

The ANOVA test helped to determine statistically significant axes. The gut microbiota had no significant effect on the innate and adaptive immune cells in the small intestine. However, the pDCs (envfit analysis, r^2^ = 0.8407, *p* = 0.013) and B cells (envfit analysis, r^2^ = 0.0.6937, *p* = 0.071) were initially linked to community structure of gut microbiota. Contrary to SH, a negative correlation between B cells and the gut microbiota occurred in SP (*p* > 0.05). In addition, pDCs were positively related to the periodontitis microbiota that colonized the small intestine (*p* < 0.05) (Figures 4A,B).

Systemic immune cells in spleen appeared to be more significant variables with respect to the gut microbiota. pDCs (*F* = 9.47, *p* = 0.001), CD11B + DCs (*F* = 5.23, *p* = 0.011), and MNPs (*F* = 3.79, *p* = 0.021) were the most important innate immune cells associated with the gut microbiota. Furthermore, pDCs (envfit analysis, r^2^ = 0.0.8515, *p* = 0.045) were positively correlated with the periodontitis microbiota that colonized the small intestine, which showed a negative relationship in SH (Figure 4C). In relation to the adaptive immune cells, gdT (*F* = 3.79, *p* = 0.021) showed a major significant factor response to the diversity of gut bacterial structure. In addition, B cells (envfit analysis, r^2^ = 0.7248, *p* = 0.094) and CD4 + T cells (envfit analysis, r^2^ = 0.7236, *p* = 0.062) were initially related to community structure of gut microbiota. Contrary to SH, there was a positive relationship between the gut microbiota and B cells in SP (*p* > 0.05). And CD4 + T cells were negatively correlated with the gut microbiota in SP (*p* > 0.05) (Figure 4D).

**FIGURE 5:**
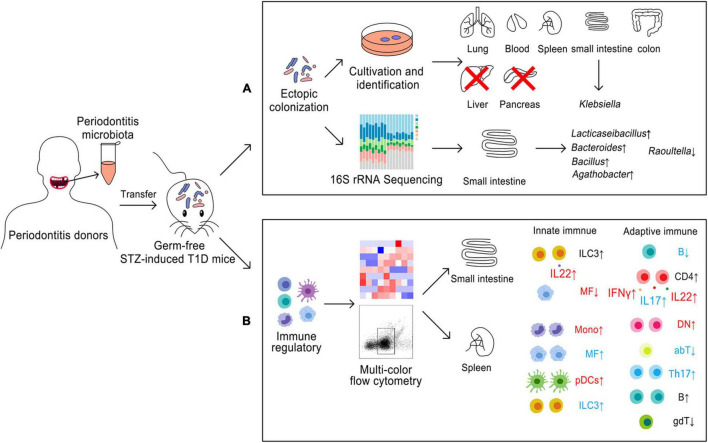
Model for ectopic colonization and immune regulation of periodontitis microbiota in germ-free mice with STZ-induced T1D. **(A)** Ectopic colonization. **(B)** immune regulation; small intestine, local immunity; spleen, systemic immunity. Blue color meant about two times alteration and red color meant about three-times changes. ↑, upregulation; ↓, downregulation.

Overall, in this study, we found that periodontitis microbiota could migrate to the distal organs, but not colonize the liver and the pancreas in the state of T1D. *Klebsiella* were the dominant genera in these systemic organs. Periodontitis microbiota also contributed to *Lacticaseibacillus*, *Bacillus*, *Agathobacter*, and *Bacteroides*, colonizing the small intestine, in contrary to *Raoultella* (Figure 5A). Innate and adaptive immune responses may be dysregulated for two or three times by periodontitis microbiota in the local or systemic organs. MFs, DN, IL22 + ILCs, Monos, IL22^+^CD4 T cells, IFNγ^+^ CD4 T cells, and pDCs might be more responsive to periodontal microbiota. Ectopic colonization of periodontitis microbiota mainly drove Th1, Th17, and Th22 cells induction and inflammation in the small intestine. pDCs might be the key immune cells which were involved in the relationship between periodontitis microbiota and T1D onset (Figure 5B).

## Discussion

In this study, we established a germ-free T1D murine model by use of STZ. A concentration of the fast plasma glucose > 8.3 mM was considered as an accurate diagnostic tool for diabetes (Furman, 2015). As in previous research (Pan et al., 2018), a model of germ-free mice with STZ-induced T1D was succeeded with the fast plasma glucose (10.55 mM), and a significant difference was shown compared to the control group in our study. However, we found that the plasma glucose, the fast insulin, and alveolar bone loss were not affected by periodontitis microbiota. Some reports showed that human gut microbiota, which was transferred to germ-free non-obese diabetic mice, would decrease the incidence of diabetes (Neuman et al., 2019). Thus, after colonization of the oral microbiota, the effect of periodontal microbiota on glucose metabolism in germ-free mice with STZ-induced T1D might be lost. Moreover, considering that it is a long-term process in the progression of β-cells damage in T1D, the immune dysregulation would be activated before the change of blood glucose metabolism. Thus, further studies should design a longer experimental period in the same animal model to determine whether colonization of periodontitis-associated microbiota could be associated with glucose metabolism and alveolar bone loss.

A model of germ-free mice with STZ-induced T1D that orally incubated with mixed saliva microbiota was used in this study. The advantage of this approach is that the model makes it possible to observe the overall impact and to provide the direct ectopic evidence of periodontitis microbiota. As the entrance of the digestive tract, the oral cavity connects to the respiratory, digestive, and external environments. Most oral microbes can colonize the intestine under several disease conditions (Gevers et al., 2014; Zhang et al., 2015; Schmidt et al., 2019). Diabetes is characterized by massive *Klebsiella* invasion, which could be directly associated with intestinal inflammation in STZ-induced diabetic rats (Wirth et al., 2014). Japanese scholars found that the oral cavity can be a reservoir for intestinal pathogens, such as *Klebsiella* (Atarashi et al., 2017). Consistent with our results, *Klebsiella* from salivary samples were the dominant genera that colonized the small intestine. *Bacteroidetes* in fecal microbiota could contribute to the development of T1D (Vaarala, 2013). Meanwhile, a low ratio of *Firmicutes/Bacteroidetes* might be an early diagnostic marker of T1D (Giongo et al., 2011). Consistent with the results in our study, the ratio of *Firmicutes*/*Bacteroidetes* was decreased in the murine small intestinal contents, where those bacteria originated from oral periodontal microbiota. An increasing intestinal permeability allows bacteria to have access to the intestinal epithelium, which is a critical step in the progression of T1D by disturbing intestinal immune response (Vaarala et al., 2008; Vaarala, 2013). Moreover, *Bacteroides* species contribute to bacterial translocation, resulting in inflammation to destroy the intestinal barrier. It was reported that *Raoultella* were negatively correlated with the hepatic lipid content (Rodriguez et al., 2020). There is a potential relationship between dysregulation of lipids metabolism and a decrease of *Raoultella* that might contribute to the progression of diabetes. By and large, periodontitis microbiota could colonize the gut and induce dysbiosis, triggering the onset of T1D.

The STZ-induced diabetic mice commonly develop with the destruction of intestinal barrier, leading to bacterial translocation to other organs (Chung et al., 2016). A risk of lung infections with *Klebsiella* is elevated by hyperglycemia. One concept of “The Oral-Lung Axis” in microbiological aspect has been proposed recently (Mammen et al., 2020; Qi and Dai, 2020). New evidence that oral *Klebsiella* tended to colonize the lung under T1D condition was provided in our study. Pancreas is considered as an aseptic environment in former studies. No bacteria are detected in the pancreas of the germ-free mice by gavage with bacterial strains (Thomas et al., 2018). However, germs can enter the pancreas by the portal vein circulation or mesenteric lymph tissue when pancreatitis or pancreatic cancer occurs (Thomas and Jobin, 2020). In our study, periodontitis microbiota could not colonize the pancreas when T1D occurred. However, the oral microbiota from the healthy subjects could translocate to the pancreas. Liver has an ability to remove gut-derived pathogens from the circulation, eradicating any translocated bacteria (Kubes and Jenne, 2018). A recent study failed to detect any bacteria in portal blood of liver under basal conditions (Balmer et al., 2014). No bacteria grew in plates, which were cultured from the liver tissues in our study. However, once liver cirrhosis developed, intestinal barrier dysfunction and pathological bacterial translocation could occur (Wiest et al., 2017). The live ectopic bacteria from periodontitis microbiota could be detected by using traditional culture-dependent method. Therefore, it requires more evidence to describe the overall ectopic ability of periodontitis microbiota by culture-independent method in the future.

The development of T1D is affected by the gut microbiota, mainly *via* altering intestinal permeability and dysregulating immune responses (Han et al., 2018). Many researchers have argued that CD4 + T cells have an important role in T1D onset (Lehuen et al., 2010; Shao et al., 2012; Ferretti and La Cava, 2016). CD4 + T cells are functionally activated by pathogens, which are mainly divided into three phenotypes, namely, Th1, Th2, and Th17. A variety of cytokines are produced by Th1 cells, such as IFNγ and IL10 (Ruterbusch et al., 2020). T1D traditionally belongs to Th1-mediated disease. An increase of IFNγ + CD4 T cells is associated with the development of T1D patients and animal model (Szablewski, 2014; Walker and von Herrath, 2016). Th17 cells are also involved in the process of T1D by producing IL-17A (Li et al., 2014; Walker and von Herrath, 2016). The levels of IL17 were elevated in the peripheral blood of T1D patients (Arif et al., 2011). Gnotobiotic studies revealed that the inappropriate colonization of certain oral commensals in the gut might trigger autoimmunity (Ruff et al., 2020). Moreover, periodontal pathogens also have an ability to induce a stronger Th1 and Th17 response (Monasterio et al., 2018; Bittner-Eddy et al., 2020). Th22 cells, a new type of characterized CD4 + T cells with IL22 as the main cytokines, are associated with the pathogenesis of autoimmune diseases (Jiang et al., 2021). A positive relationship exists between Th22 and Th17 in T1D patients and Th22 might contribute to the onset of T1D (Xu et al., 2014). Consistent with the results in our study, periodontitis microbiota could activate Th1, Th17, and Th22 lymphocytes by increasing the cytokines level of IFNγ, IL17, and IL22 in the small intestine. These cytokines might have potential pro-inflammatory ability to accelerate the progress of T1D. As a subset of T-cells, DN cells produce inflammatory cytokines and chemo-attractants to infiltrate tissues, contributing to the production of autoantibodies. The number of DN cells were increased in several autoimmune/inflammatory conditions (Mohamood et al., 2008; Crispín and Tsokos, 2009; Brandt and Hedrich, 2018). On the contrary, other studies showed that the proportion of DN cells was low in autoimmune diabetes-susceptible mice (Dugas et al., 2010). In our study, the periodontitis microbiota enlarged the accumulation of DN cells in spleen. More studies are required to clarify the role of DN cells in the progression of T1D.

Crosstalk of adaptive and innate immune cells is involved in T1D (Lehuen et al., 2010). Cytokines produced by T cells lead to recruitment of MFs. The production of TNF and IL-1β might support a pathogenic role of MFs in T1D. The frequency of MFs was decreased in the small intestine, while those cells were increased in the spleen by periodontitis microbiota in this study. More evidence is needed to clarify whether the function of MFs has been changed by periodontitis microbiota in the further study. ILC3s play an important role in the maintenance of intestinal homeostasis by generation of IL-22 and IL-17 (Zeng et al., 2019). However, the inappropriate activation of ILC3 results in overexpression of the inflammatory cytokines, such as IL-22, IL-17, and IFN-γ. Both protective and deleterious roles of ILCs in diabetes have been emerging (Miani et al., 2018). Recently, it was reported that ILC3s are the predominant subset in the periodontal tissue of periodontitis in comparison with the healthy people (Li et al., 2021). And our data showed that periodontitis microbiota helped the ILC3s to overexpress in the small intestine and spleen and the intestinal IL22 from ILCs, which might be critical to promote inflammatory immune regulation in the progression of T1D. The main function of DCs, the professional antigen-presenting cells, is to activate naive CD8 + CD4 + T cells. There mainly are two subsets of DCs, namely, the conventional or classical DCs (cDCs) and pDCs. By releasing a high level of type I IFNs, pDCs play a pathogenic role in T1D (Lehuen et al., 2010; Xia et al., 2014; Sozzani et al., 2017). Moreover, pDCs have more efficient ability to present immune complexes to T cell than cDCs in the early-diagnosed T1D patients (Lehuen et al., 2010). In our study, intestinal and splenic pDCs were positive with gut bacterial community that colonized from the periodontitis microbiota and were largely accumulated in the spleen, which might be the key immune factor in the relationship between periodontitis microbiota and T1D.

In summary, the periodontal microbiome could be widely translocated into the other organs outside the oral cavity. An imbalanced microbial community in the gut was induced by oral periodontal microbiome. A different characteristic spectrum in the local and systemic immunity was induced by the periodontal microbiome. Under T1D status, Th1, Th17, and Th22 cells induction and inflammation were driven by ectopic colonization of periodontitis microbiota in the gut. MFs, DN, IL22 + ILCs, Monos, IL22+CD4 cells, IFNγ + CD4 cells, and pDCs might be more responsive to periodontal microbiota. The activity of pDCs was regulated by gut flora that colonized from periodontal microbiota. Ectopic and immune dysregulation induced by periodontitis microbiota in the early term might contribute to the onset of T1D.

## Data Availability Statement

The data presented in the study are deposited in the Genome Sequence Archive in BIG Data Center (https://ngdc.cncb.ac.cn/gsub/), Beijing Institute of Genomics (BIG), Chinese Academy of Science repository, accession number: PRJCA007904.

## Ethics Statement

The studies involving human participants were reviewed and approved by the Ethics Committee of the West China Hospital of Stomatology, Sichuan University. The patients/participants provided their written informed consent to participate in this study. The animal study was reviewed and approved by the Ethics Committee of the West China Hospital of Stomatology, Sichuan University.

## Author Contributions

XS: methodology, data curation, data analysis, interpretation, statistical analysis, and writing—original draft, review, and editing. JL and CL: methodology and data curation. WW: writing—review and editing. BZ: data curation, data analysis, and interpretation. CY, LB, JZ, and CZ: data curation. SL: statistical analysis. HW and YL: conceptualization, funding acquisition, methodology, supervision, and writing—review and editing. All authors contributed to the article and approved the submitted version.

## Conflict of Interest

The authors declare that the research was conducted in the absence of any commercial or financial relationships that could be construed as a potential conflict of interest.

## Publisher’s Note

All claims expressed in this article are solely those of the authors and do not necessarily represent those of their affiliated organizations, or those of the publisher, the editors and the reviewers. Any product that may be evaluated in this article, or claim that may be made by its manufacturer, is not guaranteed or endorsed by the publisher.

## Footnotes

1
http://mem.rcees.ac.cn:8080
