# Supplementary material for: Ectopic Colonization and Immune Landscapes of Periodontitis Microbiota in Germ-Free Mice With Streptozotocin-Induced Type 1 Diabetes Mellitus
Source: Front Microbiol. 2022 Jun 10;13:889415. doi: 10.3389/fmicb.2022.889415 (PMC9226645; doi:10.3389/fmicb.2022.889415)
Supplement: Supplementary file 1 [file Presentation_1.pdf]

Supplementary Material

1 Supplementary methods

1.1 Fast plasma glucose and fast insulin detection

Before sacrificed, the mice were fasted for 6 h, then the blood was collected from the tail. The fast plasma glucose was analysed using an Accu-Chek performa Glucometer. A mouse insulin ELISA kit (Millipore EZMRI-13K, USA) was used to analyse the serum fast insulin. All the procedures were following the instructions.

1.2 Detection of mandibular alveolar bone resorption

Hemi-mandibles of mice were scanned using high-resolution μCT (Viva CT40; Scanco Medical, Bassersdorf, Switzerland). The parameters of μCT were at 45 keV with a 10 μm isotropic voxel size for data analysis. The alveolar bone loss (mm) was measured between the cemento-enamel junction and the alveolar bone crest. Three-dimensional reconstructions were performed using a set of 400 slices.

2 Supplementary Figures and Tables

2.1 Supplementary Figures

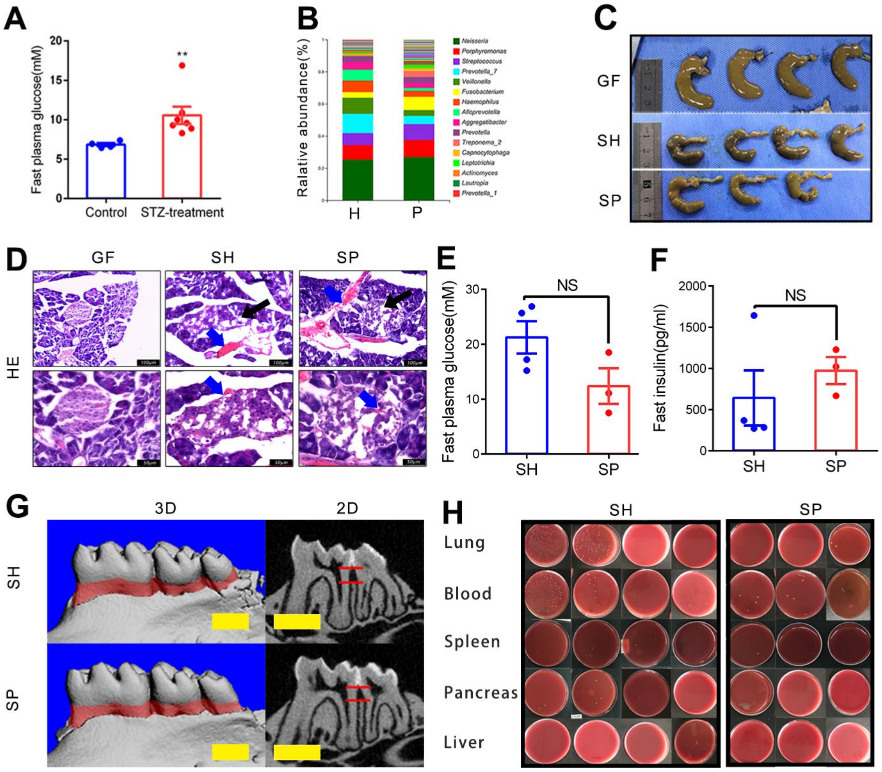

Supplementary Figure 1. The quality identification of animal model and ectopic colonization ability of periodontitis microbiota in the extra-oral sites.

(A) The fast plasma glucose of germ-free mice with STZ-induced T1D was monitored before saliva microbiota colonization. (B) The genus (of which the relative abundance ≥1% with annotation) were detected by 16S rRNA gene sequencing from the mixed saliva of periodontitis patients (P, n = 8) and healthy subjects (H, n = 12). (C) Morphology of cecum. (D) Histological analyses of pancreatic islets by hematoxylin-eosin staining. Pancreatic islets were indicated by the black arrows and vascular dilatation, congestion intra- and para-pancreatic islets were indicated by the blue ones, 200 × and 400 × magnification of images, respectively. (E-F) The fast plasma glucose and fast insulin of the mice at the endpoint experiment. (G) Hemi-mandible of 3D reconstructions and sagittal slice views of 2D by μ CT. Alveolar bone loss was described by the distance between two horizontal lines of red color. Scale bar, 1mm. (H) Systemic organ (lung, spleen, pancreas, liver) suspensions and the blood of equal quality or volume of were collected and cultured on the plates. Data were means ± SEM. Unpaired Student's *t*-tests were performed on all data. ***p* < 0.01. Control, germ-free mice (n=4); STZ-treatment, streptozotocin was injected intraperitoneally in germ-free mice (n=7).

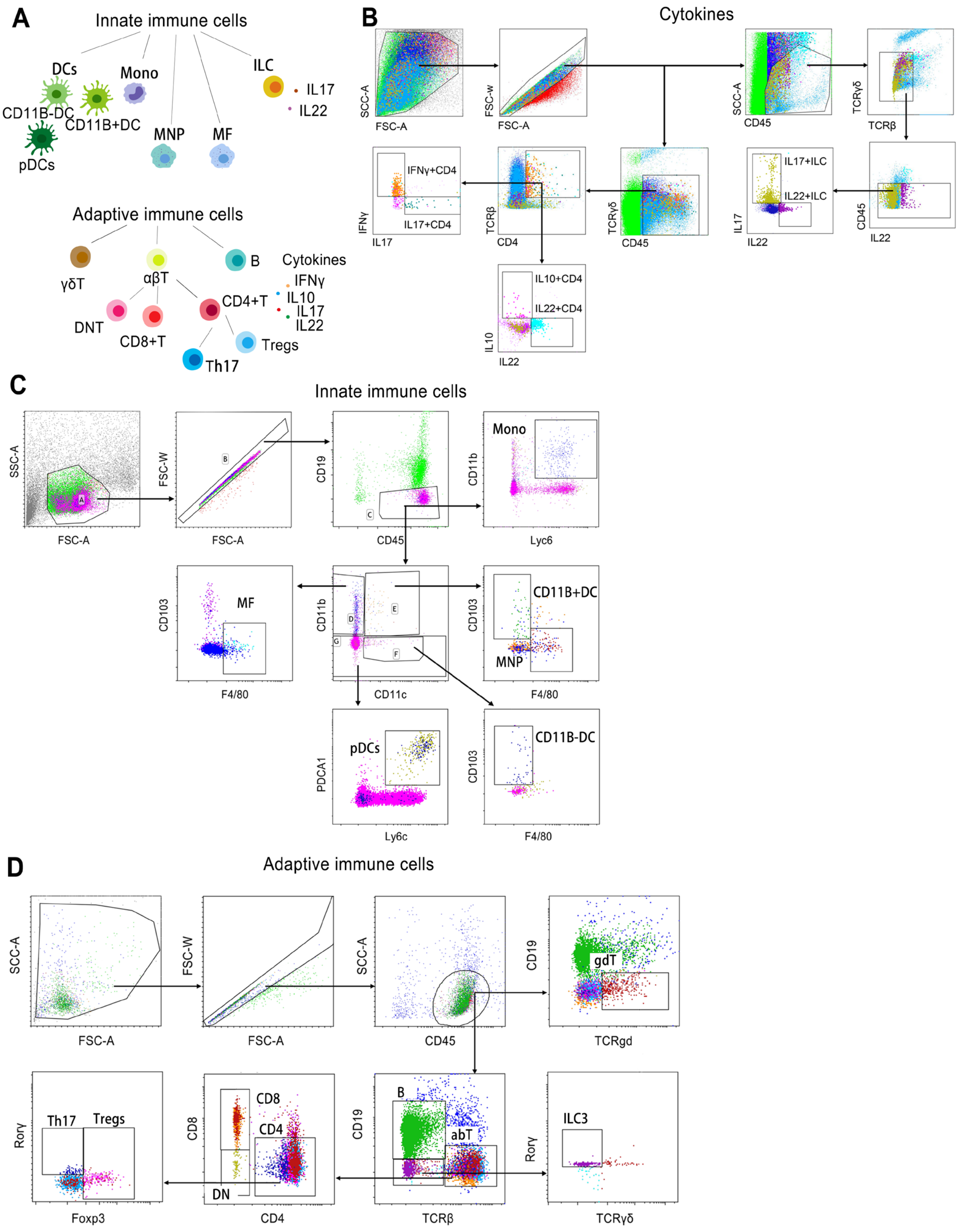

Supplementary Figure 2. Frame or gating strategy of 15 immune cells and six cytokines.

(A) 15 types of immune cell and six cytokines involved in multi-color flow cytometry. (B) The gating strategy of cytokines. (C) The gating strategy of innate immune cells. (D) The gating strategy of adaptive immune cells.

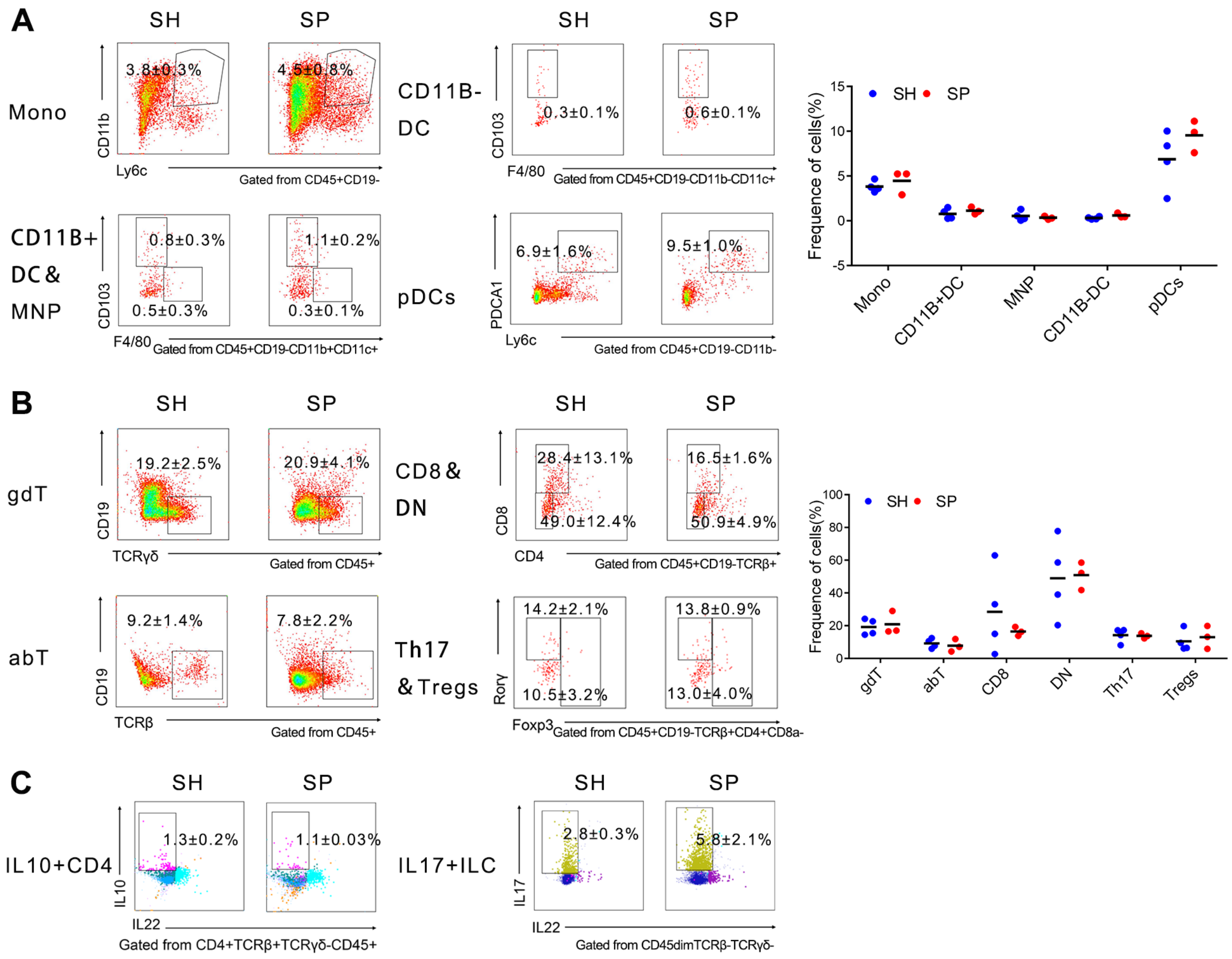

Supplementary Figure 3. Immune cells and cytokines with no significance in small intestine.

(A) Representative flow cytometry dot plots of not significant immune cells. (B) Histogram of immune cells. (C) Representative flow cytometry dot plots of not significant cytokines. Unpaired Student's t-test was analyzed all the data, which were shown as means ± SEM.

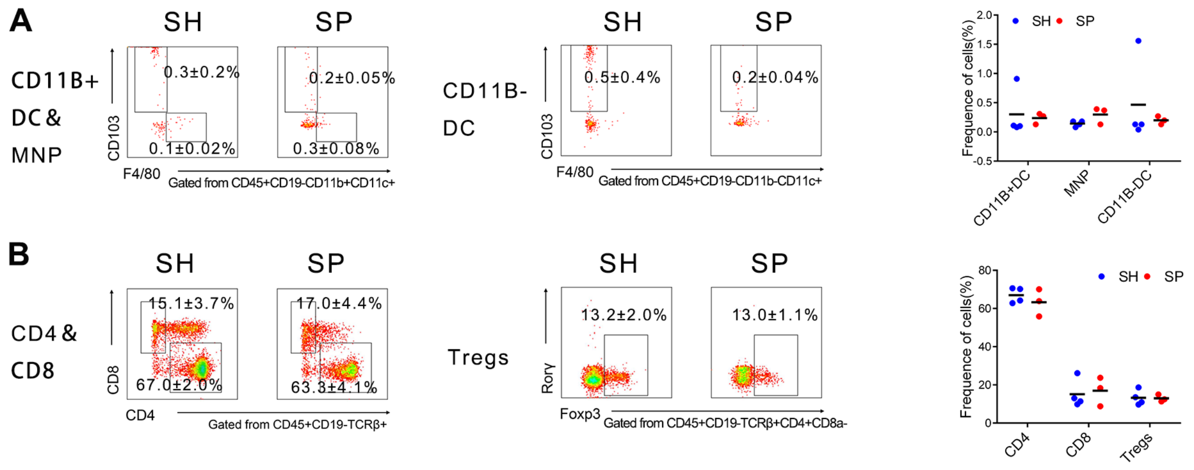

Supplementary Figure 4. Immune cells with no significance in spleen.

(A) Representative flow cytometry dot plots of no significance immune cells. (B) Histogram of those cells. Unpaired Student's t-test was analyzed all the data, which shown as means ± SEM.

Supplementary Tables

Table S1 The list of antibodies used in the multicolor flow cytometry

Antibodies	Source	Identifier
Anti-mouse CD45 Brilliant Violet 605	Biolegend	Cat#103140
Anti-mouse CD11c PE Cy7	Biolegend	Cat#117318
Anti-mouse/human CD11b Percp Cy5.5	Biolegend	Cat#101228
Anti-mouse Ly6c FITC	Biolegend	Cat#128006
Anti-mouse F4/80 Alexa 700	Biolegend	Cat#123130
Anti-mouseCD137(PDCA-1) Alexa Fluor 647	Biolegend	Cat#127106
Anti-mouse CD103 PE	Biolegend	Cat#121406
Anti-mouse CD19 APC Cy7	Biolegend	Cat#115530
Anti-mouse CD45 Pacific blue	Biolegend	Cat#103126

`Anti-mouse CD4 FITC	Biolegend	Cat#100406
Anti-mouse CD8a Alexa 700	Biolegend	Cat#100730
Anti-mouseTCRβchain PE Cy7	Biolegend	Cat#109222
Anti-mouseTCRgd Percp Cy5.5	Biolegend	Cat#118118
Anti-mouse Foxp3 APC	Affymetrix/eBioscience	Cat#17-5773-82
Anti-mouse ROR gamma(t) PE	Affymetrix/eBioscience	Cat#12-6988-80
Anti-mouse IL-17A APC	Biolegend	Cat#506916
anti-mouse IFN-γ FITC	Biolegend	Cat# 505806
Anti-mouse IL-10 Pacific Blue™	Biolegend	Cat# 505020
Anti-mouse IL-22 PE	Biolegend	Cat# 516404
Anti-mouse CD45 APC/Cy7	Biolegend	Cat# 103116

Table S2 Gating strategy of immune cells and cytokines

Cell names	Gating	Reported as % of
Monos	Ly6c+CD11b+CD45+CD19-	CD45+CD19-
MFs	F4/80+CD103-CD11b+CD11c-CD45+CD19-	CD45+CD19-
MNPs	F4/80+CD103-CD11b+CD11c+CD45+CD19-	CD45+CD19-
CD11B+DCss	CD103+F4/80-CD11b+CD11c+CD45+CD19-	CD45+CD19-
CD11B-DC	CD103+F4/80-CD11b-CD11c+CD45+CD19-	CD45+CD19-
pDCs	PDCA1+Lyc6+CD11b-CD45+CD19-	CD11b-CD45+CD19-
ILC3s	Rorg+CD45+TCRb-CD19-TCRgd-	CD45+
B	CD19+CD45+TCRb-	CD45+

gdT	TCRgd+CD45+CD19-	CD45+
abT	TCRb+CD45+CD19-	CD45+
DN	CD4-CD8a-TCRb+CD19-CD45+	TCRb+CD45+CD19-
CD8	CD8a+CD4-TCRb+CD19-CD45+	TCRb+CD45+CD19-
CD4	CD4+CD8a-TCRb+CD19-CD45+	TCRb+CD45+CD19-
Th17	Rorg+Foxp3-CD4+CD8a-TCRb+CD19-CD45+	CD4+CD8a-TCRb+CD19-CD45+
Tregs	Foxp3+CD4+CD8a-TCRb+CD19-CD45+	CD4+CD8a-TCRb+CD19-CD45+
IFNγ+CD4	IFNγ+CD4+TCRb+TCRgd-CD45+	CD4+TCRb+TCRgd-CD45+
IL17+CD4	IL17+CD4+TCRb+TCRgd-CD45+	CD4+TCRb+TCRgd-CD45+
IL10+CD4	IL10+CD4+TCRb+TCRgd-CD45+	CD4+TCRb+TCRgd-CD45+
IL22+CD4	IL22+CD4+TCRb+TCRgd-CD45+	CD4+TCRb+TCRgd-CD45+
IL17+ILC	IL17+TCRb-TCRgd-CD45dim	TCRb-TCRgd-CD45dim
IL22+ILC	IL22+TCRb-TCRgd-CD45dim	TCRb-TCRgd-CD45dim
